# Scarlet Fever Epidemic, Hong Kong, 2011

**DOI:** 10.3201/eid1810.111900

**Published:** 2012-10

**Authors:** Emma Y.Y. Luk, Janice Y.C. Lo, Amy Z.L. Li, Michael C.K. Lau, Terence K.M. Cheung, Alice Y.M. Wong, Monica M.H. Wong, Christine W. Wong, Shuk-kwan Chuang, Thomas Tsang

**Affiliations:** Centre for Health Protection, Hong Kong, People’s Republic of China

**Keywords:** Scarlet fever, epidemic, Streptococcus pyogenes, emm type, shock, septic, disease notification, antimicrobial resistance, streptococci, bacteria, Hong Kong

## Abstract

More than 900 cases of scarlet fever were recorded in Hong Kong during January–July, 2011. Six cases were complicated by toxic shock syndrome, of which 2 were fatal. Pulsed-field gel electrophoresis patterns suggested a multiclonal epidemic; *emm*12 was the predominant circulating type. We recommend genetic testing of and antimicrobial resistance monitoring for this reportable disease.

Scarlet fever is caused by infection with *Streptococcus pyogenes* and mainly affects children. An upsurge of scarlet fever occurred in Hong Kong, People’s Republic of China, in 2011, exceeding baseline annual incidence rates for the previous 2 decades. We investigated possible changes in clinical severity, transmissibility, and characteristics of the causative pathogen for this outbreak.

## The Study

Scarlet fever is a statutory notifiable disease in Hong Kong. A clinical case is defined as illness in a person who has clinical features of scarlet fever (fever and fine, sandpaper rash of characteristic distribution that blanches on pressure, with or without strawberry tongue, desquamation, or sore throat). A confirmed case is defined as a clinical case with positive throat or wound culture for *S. pyogenes* or antistreptolysin titer >200.

Epidemiologic, clinical, and laboratory data were collected by standard questionnaire for every reported case. A cluster was defined as >2 cases in persons sharing the same residential or school address within the incubation period. We compared epidemiologic, clinical, and microbiological features of the scarlet fever cases from January–July 2011 (outbreak period) with features of those reported during 2008–2010 (baseline period). We used SPSS version 14.0 (SPSS Inc., Chicago, IL, USA) for analyses; p<0.05 was considered significant.

For comparison, we performed a retrospective review of hospital discharge records kept by public hospitals. We extracted records of patients hospitalized during January 2008–July 2011 who had diagnoses that are known complications of scarlet fever, including toxic shock syndrome, acute rheumatic fever, and acute glomerulonephritis. These cases were reviewed to determine whether the complications were related to scarlet fever.

Bacterial culture of *S. pyogenes* was performed on diagnostic specimens in hospital laboratories and the Public Health Laboratory Centre of the Department of Health; the latter serves as the diagnostic and public health reference laboratory in Hong Kong. Antimicrobial drug susceptibility testing, *emm* typing, and detection of various virulence genes were performed at the Public Health Laboratory Centre on *S. pyogenes* isolates received during 2011 and archived during 2008–2010 (*1*). Pulsed-field gel electrophoresis (PFGE) was performed on the basis of the gram-positive protocol, and PFGE profiles were analyzed by using BioNumerics 5.0 software (Applied Maths, Sint-Martens-Latem, Belgium).

In June 2011, the Department of Microbiology of the University of Hong Kong announced the discovery of a unique 48-kb insertion sequence in the genome of *S. pyogenes* isolated from a blood specimen from a 7-year-old girl who died of scarlet fever (*2*). We tested for this insert in a sample of strains collected during 2008–2011 using the method provided by the University of Hong Kong.

**Figure 1:**
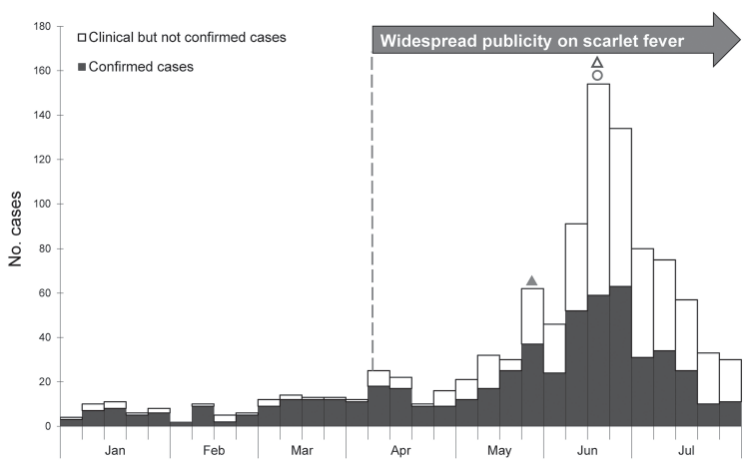
Weekly number of scarlet fever cases, by onset date, Hong Kong, January–July 2011. White bars indicate clinically diagnosed but not laboratory-confirmed cases; solid bars indicate laboratory-confirmed cases. Solid triangle indicates May 30 dissemination of press release about first fatal case (in a 7-year-old girl); open triangle indicates June 21 dissemination of press release about second fatal case (in a 5-year-old boy); circle indicates June 23 launch of health education campaign.

During January 1–July 31, 2011, a total of 996 cases of scarlet fever were reported, greatly exceeding the annual number of cases reported during 2008 (235), 2009 (187), and 2010 (128). Outbreak activity in 2011 peaked at week 26 (week ending June 25) (Figure 1). During the outbreak period (January–July 2011), the annualized incidence rate was 24.0/100,000 population, ≈9× higher than the average annualized incidence rate of 2.62/100,000 population during the baseline period of 2008–2010. During the previous 2 decades, baseline annual incidence rates ranged from 0.0351 to 3.37 cases/100,000 population.

Table 1 compares the epidemiologic features, clinical features, and laboratory results for scarlet fever cases reported during 2011 and 2008–2010. Highest incidence (547 cases/100,000 population) was reported for children 4–7 years of age (Table 1). Clinical features, complications, and case-fatality rate for cases reported in 2011 were largely comparable to those reported during the baseline period. The proportion of case-patients requiring hospitalization during 2011 was lower, and mean duration of hospital stay was ≈0.5 days shorter than for the baseline period. Details of the 9 complicated cases are shown in Table 2.

**Table 1:** Epidemiologic characteristics, clinical features, and laboratory results for scarlet fever cases reported in Hong Kong during January–July 2011 compared with cases reported during 2008–2010

Characteristic*	2008–2010, n = 550	January 1–July 31, 2011, n = 996	p value
Epidemiology			
Sex ratio, M:F	1.6:1	1.5:1	0.50
Age range (median)	9 mo–40 y (5 y)	1 mo–51 y (6 y)	0.40
Local cases	98.0 (539/550)	97.4 (970/996)	0.56
Clustering			
Cases in a cluster	5.45 (30/550)	14.4 (143/996)	<0.0001†
Cases in home clusters	3.3 (18/550); 9 clusters	6.5 (65/996); 31 clusters	
Cases in each home cluster, range (median)	2 (2)	2–3 (2)	0.34
Cases in school clusters	2.2 (12/550); 4 clusters	7.8 (78/996); 28 clusters	
Persons affected in each school cluster, no. (median)	2–4 (3)	2–7 (2)	0.42
Clinical features			
Fever	95.6 (526/548)	93.2 (928/996)	0.065
Sandpaper rash	97.4 (534/548)	95.4 (950/996)	0.13
Strawberry tongue	45.1 (248/550)	51.4 (512/996)	0.020‡
Sore throat	74.4 (409/550)	78.5 (782/996)	0.073
Desquamation	27.8 (153/550)	23.7 (236/996)	0.084
Hospitalization	63.9 (351/549)	56.6 (561/991)	0.005§
Duration of hospitalization, d (mean)	1– 25 (3.8)	1–33 (3.3)	0.005¶
Concomitant chickenpox	5.5 (30/550)	1.9 (19/996)	0.0002#
Complications**	0.73 (4/550)	0.90 (9/996)	0.79
Toxic shock syndrome	0.18 (1/550)	0.60 (6/996)	0.43
Case-fatality rate	0	0.20 (2/996)	0.54
Laboratory results			
Laboratory confirmation	46.0 (253/550)	51.8 (533/996)	0.0055††
Positive throat or wound culture	95.3 (241/253)	97.2 (521/533)	0.094
Antistreptolysin O titer >200 IU/mL	4.74 (12/253)	4.37 (12/533)	0.094

*Values are % (no./total no.) unless otherwise indicated. Lower denominators indicate data missing or not applicable. †χ^2^ = 27. ‡χ^2^ = 5.40. §χ^2^ = 7.85. ¶*t* = –2.8 (95% CI of difference 0.15–0.83 d). #χ^2^ = 14. **Complications include toxic shock syndrome, septicaemia, parapharyngeal abscess, rheumatic fever, quinsy and hepatitis. ††χ^2^ = 7.7.

**Table 2:** Clinical characteristics of patients with scarlet fever who had medical complications, Hong Kong, January–July 2011*

Characteristic	Case-patient no.								
	1	2	3	4	5	6	7	8	9
Patient age, y/sex	14/M	M/11	8/F	7/F	5/M	6/M	3/M	2/F	12/M
Month of illness onset	April	April	April	May	June	June	July	July	July
Days from symptom onset to hospital admission	1	4	7	7	4	10	1	1	0
Complications	TSS	Parapharyngeal abscess	TSS	TSS	TSS	Septicemia	TSS	TSS	Septicemia
Intensive care unit admission	No	No	Yes	Yes	Yes	Yes	No	Yes	No
Concomitant chickenpox infection	No	No	No	No	Yes	No	Yes	No	Yes
Recovered	Yes	Yes	Yes	No (died)	No (died)	Yes	Yes	Yes	Yes
*S. pyogenes* isolates	Throat	Throat	None	Blood, lower limb blister fluid	Blood and pus	Blood	Throat	Throat	Blood and pus
*emm* type	NA	NA	NA	*emm*12	*emm*1	*emm*12	NA	NA	*emm*12
48-kb insert	NA	NA	NA	+	+	+	NA	NA	–
Virulence geneprofile (*speA*¸*speB*, *speC, speF, speH, ssa*)	NA	NA	NA	−++−++	++++−+	−+++++	NA	NA	− + + + + −

*All case-patients were healthy before infection. TSS, toxic shock syndrome; NA, not available; +, positive; –, negative.

Among the 996 scarlet fever cases reported during January–July 2011, *S. pyogenes* isolates from samples from 90 patients (mostly throat swab specimens) were characterized. Strains found belonged to the following *emm* types (number and percentage of strains): *emm*12 (70, 77.8%), *emm*1 (14, 15.6%), *emm*4 (2, 2.2%), *emm*22 (2, 2.2%), *emm*2 (1, 1.1%), and *emm*3 (1, 1.1%). All strains were susceptible to penicillin, but 77 (85.6%) strains were resistant to erythromycin. Of 59 strains tested for the 48-kb insert, 78.0% (46 strains) tested positive; 39 were *emm*12 strains and 7 *emm*1 strains. Antimicrobial drug susceptibility results were available for 39 strains positive for the 48-kb insert; 3 (7.70%) were susceptible to erythromycin. Conversely, among all erythromycin-resistant *emm*12 strains in 2011 tested for the 48-kb insert, 6/42 (14.3%) yielded a negative result.

**Figure 2:**
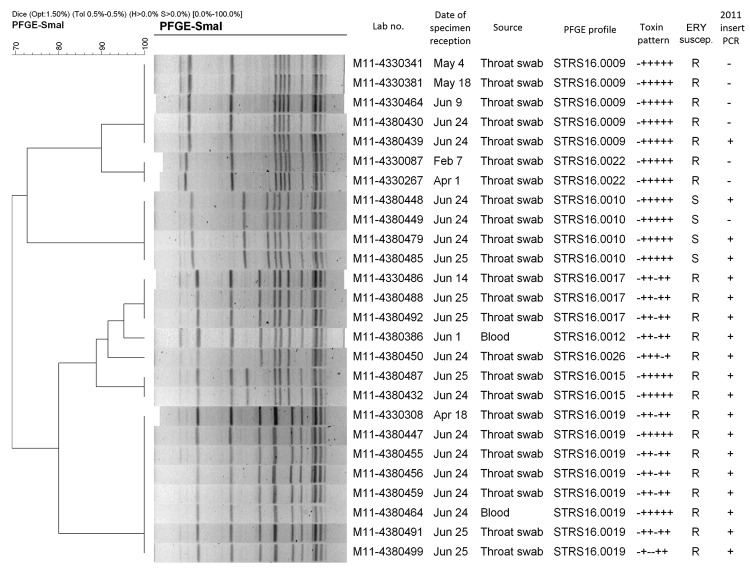
Pulsed-field gel electrophoresis patterns of 26 *emm* type 12.0 *Streptococcus pyogenes* strains, Hong Kong, 2011. Toxin profile results are shown as corresponding to the genes *speA*¸ *speB*, *speC*, *speF*, *speH*, and *ssa*. Strain M11–4380386 was from a fatal case. ERY suscep., erythromycin susceptibility result; R, resistant; S, susceptible. Scale bar indicates percent similarity.

Forty-eight *emm*12 isolates during January–June 2011 that were subjected to virulence gene profiling showed 5 virulence gene profiles. No particular virulence gene profile was dominant among the 9 scarlet fever cases associated with medical complications (Table 2). Among 26 *emm*12 strains subjected to PFGE, 7 patterns were detected; the *emm*12 strain from 1 of the 2 fatal cases exhibited a unique PFGE pattern (Figure 2). For the other fatal case, an *emm*1 strain positive for *speA* was isolated.

Of the archived *S. pyogenes* strains collected during 2008–2010, few strains were from patients diagnosed with scarlet fever; therefore, we analyzed *S. pyogenes* strains isolated from throat and superficial wound specimens from outpatients <15 years of age. Among 28 such strains, *emm*28 was detected in 9 strains; *emm*4 in 4 strains; *emm*1 in 3 strains; *emm*12, 22, and 89 in 2 strains each; and 6 other *emm* types in 1 strain each. All strains were susceptible to penicillin; the erythromycin resistance rate was 10.7% (3/28). The 48-kb insert was found in 10 (35.7%) strains: 3 strains of *emm*28, and 1 strain each of *emm* types 1, 2, 4, 22, 44, 89, and stG485.

## Conclusions

The 2011 *S. pyogenes* outbreak in Hong Kong attracted heightened media coverage, which might have increased reporting of cases; however, the higher proportion of laboratory-confirmed cases in 2011 than those during 2008–2010 suggests the upsurge was genuine. Overall clinical and epidemiologic profiles in 2011 did not differ from previous years. We found insufficient evidence that a particular *emm* type of virulence gene profile or presence of the 48-kb insert was associated with increased incidence or severity.

The reasons for the upsurge remain obscure. Laboratory findings showed diverse patterns of *S. pyogenes* strains, suggesting a multiclonal epidemic. The 48-kb insert identified in 2011 was found in *S. pyogenes* strains isolated in 2008–2010, albeit at lower rate (35.7% in 2008–2010 vs.78% in 2011). Thus, it is difficult to attribute the upsurge to the insert alone. A shift in prevailing *emm* type that occurred in 2011 might have contributed to fluctuations in the number of cases (*3*).

A higher rate of erythromycin resistance in *S. pyogenes* (>80%) was found in 2011 than in the reported previous years (20%–30%) (*4*). Because all erythromycin-resistant strains were also resistant to clindamycin (data not shown), we deduced the resistance mechanism to be resistance to macrolides, lincosamides, and streptogramins B system, as encoded by the *erm* genes (*5*).

The 48-kb insert provided a mechanism for macrolide resistance among *S. pyogenes* in Hong Kong, but our laboratory investigation found macrolide-resistant *S. pyogenes* strains and the macrolide-susceptible strains that bore them negative for this insert. Mutation of the PCR primer binding site might explain the former strains; further investigation is needed to explore this possibility.

The upsurge in scarlet fever cases in Hong Kong during 2011 likely reflects a regional phenomenon; a marked increase in cases was also observed in mainland China (*6*) and Macao (*7*) during this period. High resistance rates against macrolides were also observed for the outbreak in mainland China (*8*). We recommend close monitoring and surveillance of disease activity, genetic testing, antimicrobial susceptibility profiling, and maintaining scarlet fever’s statutory notifiable status.
